# Distinguishing the Contribution of Extracellular Electron Transfer in the *Desulfovibrio caledoniensis*-Induced Total Corrosion of Q235 Carbon Steel

**DOI:** 10.3390/ma18071613

**Published:** 2025-04-02

**Authors:** Keliang Fan, Fang Guan, Xiaofan Zhai, Guanhua Jiao, Yugang Sang, Min Jing, Jizhou Duan

**Affiliations:** 1 Department of Bioengineering, Qilu University of Technology, Jinan 250353, China; f18888260040@163.com (K.F.); jiaoguanhua2000@163.com (G.J.); 2 State Key Laboratory of Advanced Marine Materials, Institute of Oceanology Chinese Academy of Sciences, Qingdao 266071, China; weijialiu03@gmail.com (Y.S.); duanjz@qdio.ac.cn (J.D.); 3 Guangxi Key Laboratory of Marine Environmental Science, Institute of Marine Corrosion Protection, Guangxi Academy of Sciences, 98 Daling Road, Nanning 530007, China; 4 Department of Materials Science and Engineering, Qilu University of Technology, Jinan 250353, China; 5 College of Materials Science and Engineering, Shandong Jianzhu University, Jinan 250101, China; shandajingm@163.com

**Keywords:** microbially influenced corrosion, electrical microbially influenced corrosion, carbon source, sulfate-reducing bacteria, electrochemical mechanism

## Abstract

Microbially influenced corrosion (MIC) in anaerobic environments accounts for many severe failures and losses in different industries. Sulfate-reducing bacteria (SRB) represent a typical class of corrosive microorganisms capable of acquiring electrons from steel through extracellular electron transfer processes, thereby inducing severe electrical microbially influenced corrosion (EMIC). Although prior research has underscored the significance of extracellular electron transfer, the contribution of EMIC to the whole MIC has not been comprehensively studied. In this study, Q235 steel coupons were employed in an H-shaped electrochemical cell to conduct electrochemical and coupon immersion experiments, aiming to determine the contribution of EMIC to the overall MIC. The experiments were conducted under two distinct carbon source conditions: 100% carbon source (CS) and 1% CS environments. It was observed that the biotic electrodes exhibited significantly higher cathodic currents, with the most pronounced biological cathodic activity detected in the 100% CS biotic medium. The voltammetric responses of the electrodes before and after changes in the medium confirmed the biocatalytic capability of the attached biofilm in stimulating the cathodic reaction. The proportion of EMIC in MIC was calculated using linear polarization resistance, revealing a trend over time. Additionally, weight loss tests indicated that the contribution of EMIC to the total MIC was approximately 27.69%. Furthermore, the results demonstrated that while the overall corrosion rate was lower in the 1% CS environment, the proportion of EMIC in MIC increased to approximately 37.68%.

## 1. Introduction

Microbiologically influenced corrosion (MIC) is a major cause of failures in engineering materials. MIC was discovered more than 100 years ago [1]; it is believed to account for ~20% of the corrosion of metals and building materials [2,3,4]. Further, 50% [5] of all pipeline failures and 40% [6] of all pipeline internal corrosion in the gas industry can be attributed to MIC. Sulfate-reducing bacteria (SRB) are generally considered to be the main originators of MIC in marine facilities, such as harbor-wharf steel piles, seabed pipelines, and oil platforms, which are susceptible to severe corrosion [7]. Owing to their high-intensity and high-toughness properties, Q235 steels are widely employed for the long-distance transportation of oil and gases [8].

Sulfate-reducing bacteria (SRB) represent a typical class of corrosive microorganisms capable of acquiring electrons from steel through extracellular electron transfer processes, thereby inducing severe electrical microbially influenced corrosion (EMIC). In addition, the metabolites of SRB could also attack iron chemically inducing chemical microbially influenced corrosion (CMIC) of steel [9]. Dinh et al. [10] revealed that SRB accelerate the cathodic reaction to promote metal corrosion through metabolites, such as hydrogen sulfide generated by SRB (Fe + H_2_S → FeS + H_2_). Since SRB generally consumes organic compounds, the net reaction of CMIC will be expressed as follows [10]:

(1) $$6\left[{\mathrm{CH}}_{2}O\right]+4\mathrm{Fe}+4SO_{4}^{2-}+2H^{+}\to 6HCO_{3}^{-}+4FeS+{4H}_{2}O$$

During EMIC, the sessile cells in a biofilm employ an energetic metal, such as elemental iron, as an electron donor, which is expected to directly and effectively accelerate the corrosion. The resulting net reaction of direct corrosion is as follows:

(2) $$4\mathrm{Fe}+SO_{4}^{2-}+4H_{2}O\overset{SRB}{\to }FeS+3{\mathrm{Fe}}^{2+}+8OH^{-}$$

EMIC applies to electrogenic biocorrosion [11], while CMIC can occur in most microorganisms.

The research conducted by Xu Dake’s [4,12] team demonstrated that SRB cell preferentially utilizes an organic carbon as the electron donor when there are sufficient carbon sources (CSs) in the environment. Moreover, Extracellular Electron Transfer (EET) is not required since the oxidation of organic carbon and the reduction in sulfate occur in the bacterial cytoplasm [13]. However, EET is required when Fe is utilized [14], especially when the organic carbon sources in the environment are limited.

The EET depends on an elaborate electron-transfer system to transport electrons across cell walls. The electrons that are obtained via the oxidation of metals or organic carbon were transferred to cytochrome c on the periplasm before they participate in redox reactions [15]. Electrically active SRB cells yielded electrons and accelerated metal corrosion via direct electron transfer (DET) or mediated electron transfer (MET) pathways [16].

DET employs the membrane-bound enzyme complex (mainly cytochrome c) or electrically conductive nanowires of the bacteria for electron transfer. The cytochrome protein contains multiple hemes, and the electrons can jump between the Fe atoms of the adjacent hemes, thereby ensuring that the protein could conduct electrons. Gu et al. [17] proposed the mechanism for the biological cathodic catalysis to reduce sulfate based on bioenergetics. Therein, SRB probably acted as the biocathode in the corrosion process. Further, the formation of putative nanowires was observed on carbon steel in the absence of the CS [18], which was responsible for the acquisition of energy from the carbon steel and the maintenance of the bacterial energy metabolism. Deng et al. [19] demonstrated that *Desulfovi brioferrophilus* strain IS5 could respire with extracellular solids by the direct uptake of electrons via the outer membrane cytochromes [20]. Moreover, those outer membrane cytochromes were highly expressed on the surface of the cells and nanofilaments to respond to the limitation of the electron donor, indicating that *D. brioferrophilus* IS5 accelerated the anaerobic corrosion of Fe via direct electron uptake from solid minerals in energy-poor marine.

MET depends on soluble carriers to transport electrons between a biofilm and electrodes. Several studies [21,22,23,24] confirmed the significant role of the electron-transfer media in MIC, indicating that the electron transfer process was the bottleneck of *Desulfovibrio vulgaris*-induced corrosion. Additionally, more efficient EET was achieved via the addition of electron mediators to the solution [25]. Scott H. Saunders et al. found that [26] demonstrated that the secreted electron mediators could be retained to mediate efficient EET among biofilms, thus enabling the redox cycling of the electron shuttles without losing them in the biofilm.

MIC involves the interaction between materials and microbes; it is accompanied by a series of chemical reactions and the formation of corrosion products [27,28]. However, the mechanisms of MIC vary with different metallic materials, bacterial strains, and environments. The corrosion of carbon steel caused by *Desulfovibrio vulgaris* (*D. vulgaris*) is mainly EMIC [29], while CMIC dominates that of copper alloys [30]. The study of corrosion due to *Desulfovibrio caledoniensis* (*D. caledoniensis*) revealed that EMIC and CMIC were the most probable corrosion mechanisms of the Al–Zn–In–Cd [31] and 5052 aluminum alloys, respectively [32]. However, only a few studies have reported the proportion of EMIC or CMIC in the MIC of metals.

Thus, summarizing the results of the previous studies, it was assumed that EMIC and CMIC might occur concomitantly throughout the whole MIC process induced by electroactive microorganisms. While EMIC and CMIC play different roles in MIC under different environmental conditions.

This study is aimed at gaining improved insight into the MIC of Q235 steel due to *D. caledoniensis*, as well as the significance of the attached biofilm on MIC, which encompasses the following aspects: (1) the proportion of EMIC in the whole MIC; (2) the change in the proportion with time, and (3) the change in the proportion when the SRB cells were exposed to different levels of carbon starvation. To distinguish EMIC from CMIC, a 0.22-µm filter membrane was introduced into an H-shaped electrochemical cell to prevent the passage of bacteria while allowing the metabolites to permeate. In this study, the differences in the EMIC ratios of the whole MIC availed new interpretations of the underlying EET mechanisms, as well as the interaction between *D. caledoniensis* and metal corrosion.

## 2. Materials and Methods

### 2.1. Strain, Growth Medium, and Culturing Conditions

In this study, the employed *D. caledoniensis* was obtained from the rust layers of carbon steel [33]. Previous studies demonstrated that a *D. caledoniensis* biofilm could conduct electrons from a polarized electrode [34,35]. Further, all the utilized solutions and culture media were prepared from analytical-grade chemicals. *D. caledoniensis* was cultured anaerobically in 100 mL penicillin bottles in a sterile Postgate C (PGC) medium [36], as well as an N_2_ headspace. The penicillin bottles were sealed and cultured at 30 °C after incubation with full carbon sources (100% CSs) and 1% CSs. In the starvation tests, 1% CSs was achieved by modifying the full medium minus 99% lactates and yeast creams, and the 100% CS condition (full medium) was employed as the control.

### 2.2. Electrochemical Cell and Bacterial Inoculation

**Scheme 1:**
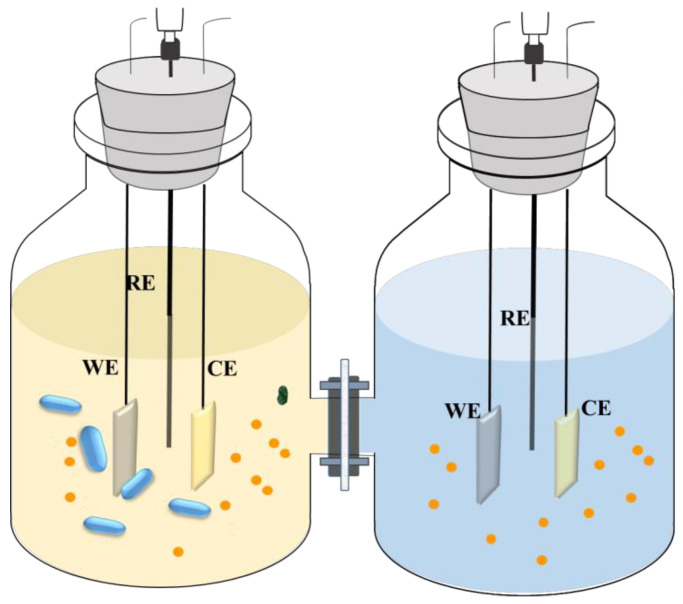
Schematic diagram of the H-shaped electrochemical cell. CE is Counter Electrode, WE is Working Electrode, RE is Reference Electrode. The balls are metabolites and the zeppelins are SRB cells.

The electrochemical measurements were conducted in the H-shaped electrochemical cell included by two 500 mL bottles (Scheme 1). Each bottle was filled with the PGB medium and accommodated three electrodes. Q235 steel coupons [37] were employed to prepare the working electrode, as previously described [38], while a platinum plate and a saturated calomel electrode (SCE) served as the counter and reference electrodes, respectively. A 0.22-µm filter membrane was mounted between the two bottles. The electrochemical cell and electrodes were sterilized before the reactor was assembled. Next, a 1% inoculation of 5-day-old bacteria cell inoculum was injected into one of the bottles containing the H-shaped electrochemical cell, and the initial concentration of the cells was ~106 cells/mL [37]. In this study, four conditions were investigated employing different combinations of CS levels and bacterium additions in the PGB solution: 100% CS with SRB; 100% CS without SRB; 1% CS with SRB; and 1% CS without SRB. The reactor was kept at a constant temperature of 30 °C. After the incubation in different culture media for 15 days, the media were replaced by a sterile, deoxygenated phosphate-buffered saline (PBS)-based medium [39].

### 2.3. Electrochemical Measurements

Electrochemical impedance spectroscopy (EIS), linear polarization resistance (LPR), and LSV were conducted with an electrochemical analyzer Gamry (interface 1000; Warminster, PA, USA). The open-circuit potential (*E*_ocp_) shift was monitored and controlled at <5 mV in all the cases before the EIS and LPR measurements [40,41]. The parameters for the measurements were selected, as follows: EIS (frequencies, 10^5^–10^−2^ Hz; sinusoidal perturbation, 5 mV) and LPR (scan rate, 0.33 mV/s, scanning range: −5 to 5 mV vs. *E*_ocp_. After the immersion for 15 days, the LSV was performed over a range of ΔE = −450 mV at a scan rate of 1 mV/s, starting from *E*_ocp_.

### 2.4. Weight Loss Determination

The weight losses of steels in different environments were determined from the difference in weights before and after removing corrosion products, following the standard practice ASTM G1-03 [42]. The corrosion rates were calculated according to the following formula [43]:

(3) $$V=\frac{8.76\times 10^{4}\times ∆m}{\rho At}$$

where V, Δm, ρ, and A are corrosion rate (mm/y), weight loss (g), coupon density (g/cm^3^), and exposed coupon area (cm^2^), respectively, while t refers to the incubation time (h) in this study.

## 3. Results

### 3.1. Corrosion of the Q235 Steel in Different Culture Media

**Figure 1:**
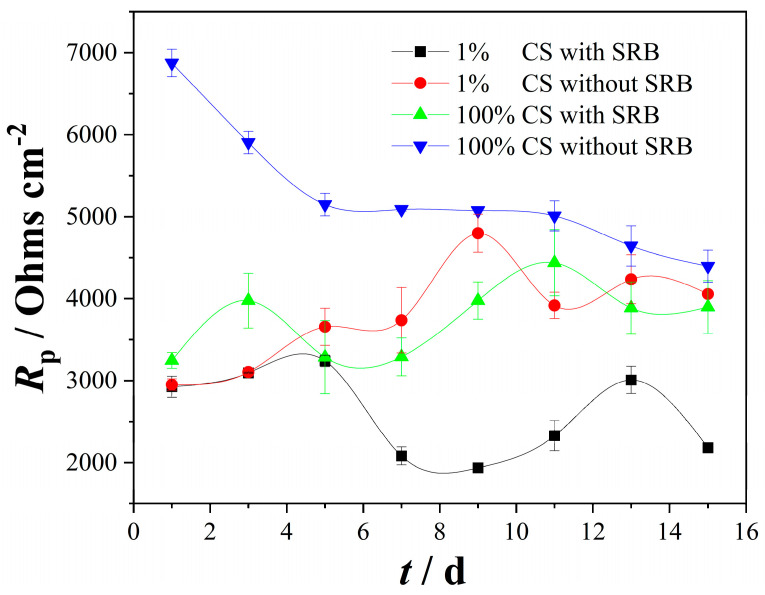
Temporal variation of *R*_p_ values for Q235 steel in different SRB environments under different carbon source concentrations (100% CS and 1% CS). (The EIS tests were performed independently in duplicates, and the error bars represent the standard deviations).

The values of the polarization resistance (*R*_p_) were measured via the LPR tests during the 15-day incubation under different conditions. Figure 1 shows the changes in *R*_p_ in the culture media with the exposure time. On the first day, the highest *R*_p_ was observed in the system of abiotic 100% CS, followed by that biotic 100% CS. The lowest *R*_p_ were found in both abiotic and biotic 1% CS system. In the abiotic 100%-CS media, *R*_p_ decreased significantly with the exposure time during the incubation, while in the 100%-CS media inoculated with SRB, the *R*_p_ fluctuated with immersion time, which may be caused by the SRB metabolic activity and the process of deposition and destruction of corrosion products.

A slight increase on *R*_p_ was observed during the first 5 days for the SRB-inoculated coupon in the 1%-CS medium, followed by a decrease and fluctuation in the last 10 days. The *R*_p_ in the sterile medium without SRB fluctuated in a small range and generally exhibited an upward trend with time. On day 15 of immersion, the highest *R*_p_ was observed for the coupons in the sterile 100%-CS culture medium, and the lowest *R*_p_ was observed for the coupons in the SRB-inoculated 1%-CS medium, while the *R*_p_ in sterile 1%-CS medium and SRB-inoculated 100%-CS culture were similar. The results demonstrated that the steel in the 1% CS SRB-inoculated solution was more susceptible to MIC compared to that in the 100% CS solution. This finding aligns with previous studies, indicating that under moderate CS starvation conditions, SRB cells may alter their energy acquisition strategy, relying more heavily on electron uptake from steel to sustain survival [12].

EIS was measured in the electrochemical glass cells after the culture media were changed by sterile and deoxygenated PBS, excluding any influence from the depletion of the soluble redox mediators, a lack of other nutrients, and/or pH change in the solution. Thus, the difference between the EIS measurements in the abiotic and biotic environments under the same CS concentration was mainly caused by the attached biofilm. Thus, the role of the attached biofilm in the MIC and electron transfer could be determined by comparing the EIS measurements in the two environments. Moreover, starved SRB-inoculated biofilms were expected to obtain energy from the steel to conserve energy [29]. If the 1%-CS SRB solution having a more severe MIC than that of the 100% case was caused by the more efficient electron uptake by the attached bacterial cells, it would be evident via the smaller magnitude of the impedance, as well as highly efficient electron transfer in the 1% CS than in the 100%-CS SRB media.

**Figure 2:**
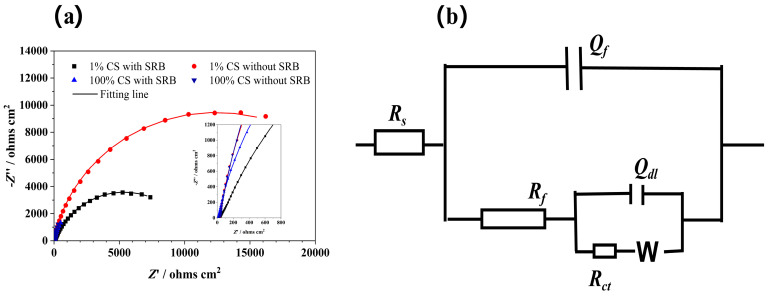
(**a**) Nyquist plots of Q235 steel in sterile and deoxygenated PBS solution after 15 days’ immersion in SRB environments with different carbon source concentrations (100% CS and 1% CS), (**b**) The equivalent circuit used to fit the EIS spectrum.

Figure 2a shows the Nyquist plots of the Q235 steel in the sterile and deoxygenated PBS solution after immersion in different SRB solutions for 15 days; the experimental data (scatter dot) is consistent with the fitting line (straight line). The impedances, represented by the diameter of the semicircle, were significantly higher in the sterile media than in the SRB-inoculated media under the same concentration of CS, indicating that the biofilm decreased the corrosion resistance. The trend in the corrosion resistance correlated with that before the change in the media. In the sterile media, the impedance magnitude improved with the decreasing CS concentration. This might be caused by the difference in the corrosion deposits or layers that were formed on the surfaces of the steel. Bacterial metabolites might permeate the membrane and participate in the reactions with the electrodes. However, the largest and smallest impedance diameters were obtained for the coupons in the 1%-CS sterile and 100%- CS SRB media, respectively. Since the EIS measurements were conducted in the same PBS solutions, the difference in the impedances was caused by the attached biofilm and corrosion deposits on the surfaces of the electrode. Iron sulfide (FeS) has been reported to mediate electron transfer and induce severe metal corrosion [44] or protect metals from rapid corrosion [45].

The impedance spectra (Figure 2b) were fitted with equivalent electrical circuits employing a two-time constant circuit model [46,47]. *R*_s_ is the solution resistance. A constant phase element (*Q*) was employed instead of capacitance. *Y* and *n* are constant phase element parameters. Thus, *Q*_f_ and *R*_f_ represent the capacitance and resistance of the corrosion product and biofilm, respectively; *Q*_dl_ is the double-layer capacitance; and *R*_ct_ is the charge-transfer resistance. The electrochemical kinetic parameters are listed in Table 1, and the results demonstrated that the values of Rs in all the electrochemical cells were similar. The abiotic coupons exhibited significantly higher *R*_ct_ values compared to the coupons containing the SRB biofilm at the same CS concentration, indicating an increased charge transfer between the metal surface and biofilm, thus proving the facilitating effect of the attached biofilm in the MIC and electron transport. Regarding the biotic coupons containing the SRB biofilm, the steels in the 100%-CS medium exhibited a smaller *R*_ct_ value than that in the 1%-CS medium. Similarly, regarding the abiotic coupons, those in the 100%-CS medium exhibited a much smaller *R*_ct_ value than 1%-CS media. The difference in the *R*_ct_ values of the abiotic coupons might correspond to the formation of a conductive ferrous sulfide layer [48].

### 3.2. Quotient of EMIC in the MIC of the Q235 Steel

The corrosion current density can be obtained via the following empirical formula:

(4) icorr=BRp

where B (mV) is the empirical constant (26 mV) [49].

Regarding the coupons that were exposed in the SRB culture media, the steels suffered chemical attacks from the bacterial metabolites, such as H_2_S, and the direct uptake of electrons from the metals via the biofilm. Thus, the MIC of the biotic coupons comprised CMIC, EMIC, and the inherent corrosion in the aqueous solution. The current density of the corrosion of the steel in the SRB culture media (*I*_corr(SRB)_) can be defined, as follows:

(5) $$I_{corr(SRB)}=I_{0}+I_{corr(CMIC)}+I_{corr(EMIC)}$$

where I_0_ represents the inherent corrosion current of the steel in the solution and *I*_corr(CMIC)_ and *I*_corr(EMIC)_ represent the corrosion currents of the steel due to the CMIC and EMIC mechanisms, respectively.

However, the abiotic and inoculated culture media were separated by the 0.22-µm membrane, which allowed only the passage of the metabolites while blocking that of the bacteria cells. Thus, the corrosion current density of the abiotic coupons comprised CMIC and the inherent corrosion, as expressed via the following equation:

(6) $$I_{corr\left(\mathrm{no}\;\mathrm{SRB}\right)}=I_{0}+I_{\mathrm{corr}(CMIC)}$$

Therefore, a partition coefficient (the quotient) or contribution of EMIC to the whole MIC can be expressed, as follows:

(7) $$Q_{\left(EET-MIC\right)}=\frac{I_{corr(\mathrm{EMIC})}}{I_{corr\left(\mathrm{SRB}\right)}}\times 100\%=\frac{\left[I_{corr\left(\mathrm{SRB}\right)}{-I}_{corr\left(\mathrm{no}\;\mathrm{SRB}\right)}\right]}{I_{corr\left(\mathrm{SRB}\right)}}\times 100\%$$

where *Q*_(EMIC, 100% CS)_ and *Q*_(EMIC, 1% CS)_ represent the quotients of *I*_corr(EMIC)_ in the total corrosion current (*I*_corr_) at 100% and 1% CS concentrations, respectively.

**Figure 3:**
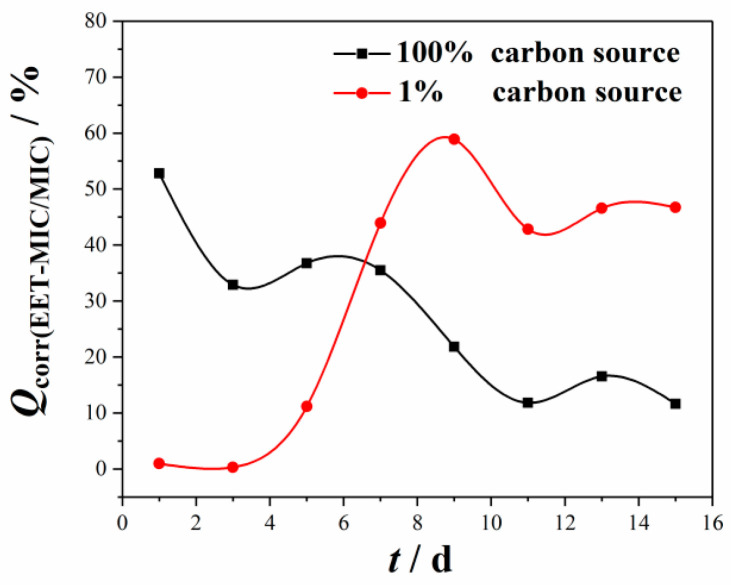
Temporal variation in Q_(EMIC)_ for Q235 steel induced by *Desulfovibrio caledoniensis* in environments with varying carbon source concentrations (100% CS and 1% CS).

Figure 3 shows the change in *Q*_(EMIC)_ at different CS concentrations with time. *Q*_(EMIC)_ fluctuated with time and varied with the CS concentration of the solution. It was found that the value of *Q*_(EMIC, 100% CS)_ was higher during the first 5 days and then much smaller in the last few days than *Q*_(EMIC, 100% CS)_. *Q*_(EMIC, 100% CS)_ fluctuated and showed a downward trend during the 15-day experiment period. The value of *Q*_(EMIC, 100% CS)_ was 52.8% on the first day, 32.8% on the third day, and 11.6% on the last day during the experiment period. The results obtained in the 100%-CS SRB culture medium indicated that CMIC was crucial to the total MIC of the metal during the whole 15-day experiment, and its effect increased gradually over time probably due to the accumulation SRB metabolic products. However, for the coupons in the 1%-CS SRB culture medium, *Q*_(EMIC, 1% CS)_ increased rapidly during the first 9 days, reaching a value of 58.9% on the 9th day, followed by a decline in the last few days of incubation. The results obtained in the 1%-CS SRB solution are attributable to the low concentrations of the cells at the beginning of incubation owing to the low CS concentration, thus causing a slight difference between the corrosion rates in the sterile and bacterial environments. The difference between 1%-CS biotic and abiotic coupons increased with the attachment of SRB cells on steels and its accompanying metabolic activity. The results indicated that the MIC type varied with different CS concentrations and was controlled by the metabolic activity of the bacteria and soluble aqueous environment.

### 3.3. Weight Loss

**Figure 4:**
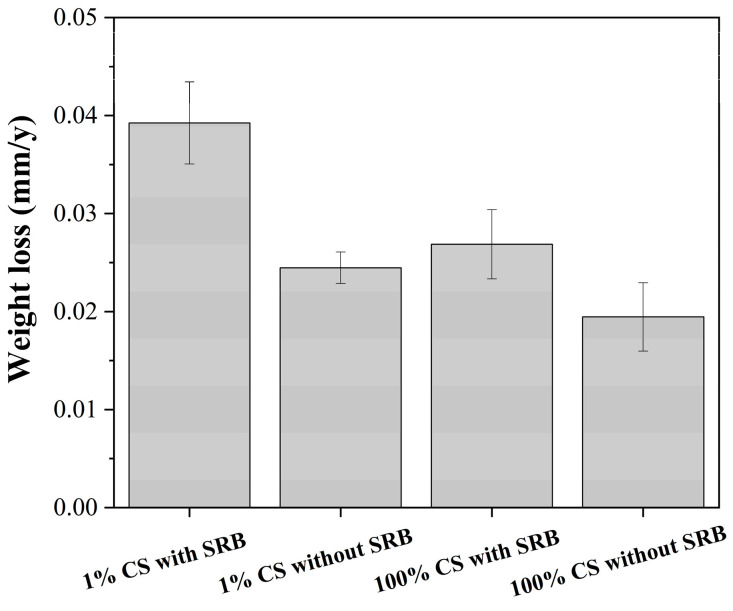
Weight losses of Q235 steel coupons in 100% CS and 1% CS media with or without SRB over a 15-day immersion period. (Error bands represent the standard deviation of at least three independent replicate samples).

The average weight loss data after the 15-day incubation period under different conditions are listed in Figure 4. For the coupons in SRB inoculated media, the weight loss in 1% CS media was much higher (0.039 ± 0.004 mm/y) than that in 100% CS media (0.027 ± 0.003 mm/y) (*p*-value = 0.001), which was consistent with the previous results [29]. The weight loss results showed a significant difference (*p*-value = 0.016) between the corrosion rate in 100% CS biotic and abiotic media (0.019 ± 0.003 mm/y). The corrosion rate of specimens in 1% CS biotic medium was 1.60 times higher than that in abiotic medium (*p* = 0.016). However, there were no significant differences between the coupons in 100% and 1% CS media without SRB inoculation (*p*-value = 0.075), although the weight loss results showed that the weight loss of coupons in 100% CS (0.019 ± 0.003 mm/y) was lower than that in 1% CS sterile media (0.24 ± 0.004 mm/y). The interesting phenomenon may be caused by the higher yeast concentration of in 100% CS than that in 1% CS solution, as the absorption of yeast on coupon surfaces may inhibited the further corrosion of metal in seawater [50,51].

A 0.22-µm filter membrane was mounted between the abiotic and biotic system to find the difference in corrosion rate between CMIC and EMIC. So, the mean partition coefficient (quotient) or contribution of EMIC to total MIC during the experiment period can be formulated as:

(8) $$Q_{mean\left(EMIC\right)}=\frac{V_{SRB}-V_{no\;SRB}}{V_{SRB}}\times 100\%$$

where V_SRB_ and V_no SRB_ are the corrosion rate calculated from weight loss of coupons cultured with and without SRB inoculated, respectively.

**Figure 5:**
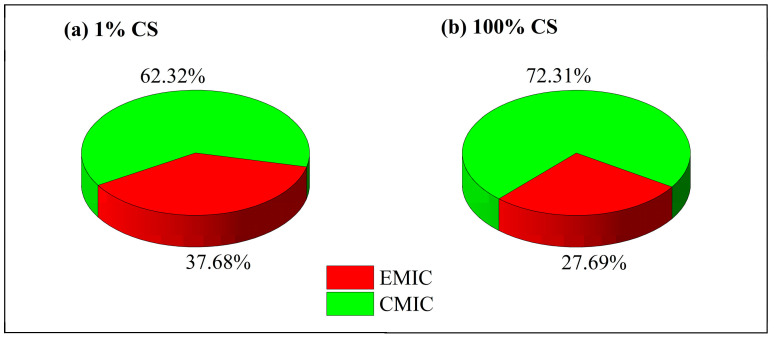
The quotient of EMIC to the total MIC of Q235 steel induced by *Desulfovibrio caledoniensis* in (**a**) 100% CS and (**b**) 1% CS media.

The *Q*_mean(EMIC)_ in 100% and 1% CS media were obtained via Formula (7) and shown in Figure 5. The quotient of EMIC to the total MIC is 27.69% and 37.68% in 100% and 1% CS media, respectively. It is not surprising to find that the *Q*_mean(EMICT)_ increased when the carbon sources are limited. However, it was interesting to find that EMIC did not dominate in either 100% or 1% CS system.

### 3.4. Revealing the Cathodic Microbial Activity via LSV

After the incubation in different culture media for 15 days, the electrodes were transferred into the sterilized, deoxygenated PBS solution. Thus, all the measurements were conducted in electrolytes of similar compositions, which excluded the influence of the compositional changes in the electrolyte during the incubation. The contributions of the activity of the attached bacteria or deposited iron sulfides in catalyzing the cathodic current could be distinguished by employing the LSV curves. The LSV measurements were conducted before and after the media change at a selected scan rate (1 mV/s), which was sufficiently slow to ensure that the steady-state of the attached biofilm was achieved [52,53,54].

After transferring the electrodes from the different culture media into sterile PBS solutions, different degrees of differences were observed in the current responses of the cathodic currents. The replacement of the medium removed the planktonic cells and possible electron shuttles when the biofilm that was formed on the electrodes was moved from the SRB media into the PBS solution. Thus, for the SRB-cultured steels, the difference in the cathodic current responses before and after the media change reflected the contributions of the planktonic *D. caledoniensis* cells and soluble electron transfer mediators to the cathodic currents. Although for the steels that were cultured without SRB, the decreases in the currents before and after the changes in the media reflected the contribution of the soluble electron-transfer mediator, which could penetrate the membrane. This current pattern revealed the contribution of the suspended *D. caledoniensis* cells and/or soluble electron-transfer agents to the generation of current.

**Figure 6:**
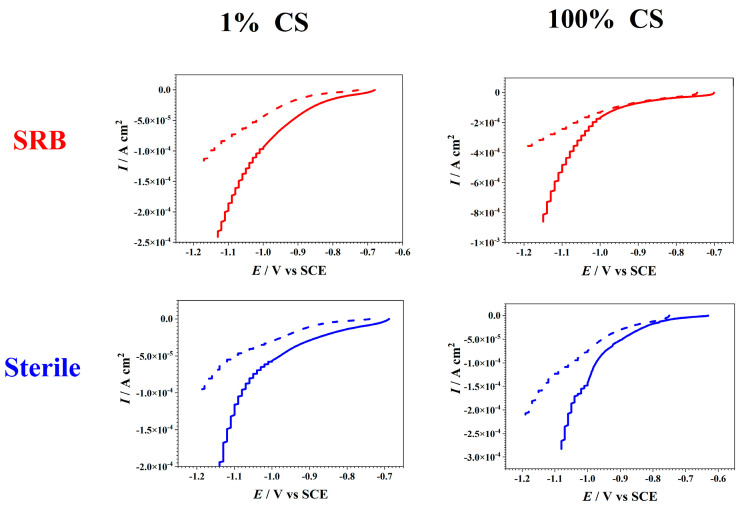
Cathodic current densities (i) vs. applied potentials of the electrodes that were incubated in biotic (red) and abiotic (blue) culture media with different CS sources for 15 days. The measurements were conducted before (solid lines) and after (dash lines) media change.

After the culturing in SRB media for 15 days, the electrodes were covered with the biofilm and corrosion products, and there was a distinguishing difference in the cathodic currents of all the coupons before and after the changes in the media, i.e., evident cathodic biological activity (Figure 6) [55]. The current drop was also observed in the cathodic currents in the abiotic environments before and after the replacement of the solution. The current drop in all coupons before and after media change reflected the contribution of MET in catalyzing current production. The current response in the 100%-CS SRB was higher than that in the 1% one when the measurements were conducted in identical electrolyte, indicating the increased electrocatalytic activity. The most significant cathodic current of the electrodes in the 100%-CS SRB medium might be caused by the biological activity of the biofilm, which conducted electrons from the electrode [18]. However, the modest cathodic biological activity in the 1%-CS media might be attributed to the low number of cells caused by the reduced concentration of CS. Since no biofilm was formed on the surface of the electrode in the aseptic environment, the difference in the current response before and after media change could be attributed to the differences in the soluble mediators and corrosion products on the electrodes. The smallest cathodic current was observed in the 1%-CS media, and it could be attributed to the limitation of the CS.

**Figure 7:**
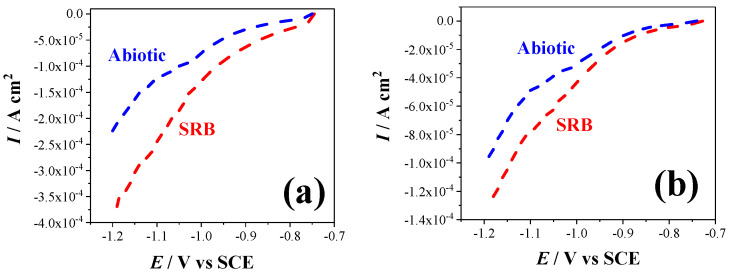
Comparison of the voltammograms of the electrodes in the sterile, deoxygenated PBS after the incubation in the (**a**) 100%-CS culture medium and (**b**) 1%-CS culture medium with or without SRB for 15 days.

The electrodes that were cultured in the SRB-inoculated media were covered with the biofilm and a black precipitate, whereas those in the abiotic culture medium did not exhibit any biofilm. Here, it was assumed that the contributions of the corrosion products, such as iron sulfide on the surface of the electrode, to the cathode current was negligible compared with that of the biofilm [55]. Thus, the more significant cathodic currents of the biotic electrodes than those of the abiotic electrodes might be attributed to the cathodic biological activity of the attached biofilm (Figure 7). The results demonstrated that the significant differences between the cathodic currents before and after the changes in the media were observed in both the 100%- and 1%-CS media, respectively. These results indicated that the biofilm that was formed on the surface of the electrode in the 100%-CS media might exhibit higher bacterial activity and mediate the cathodic current more efficiently compared with those in the 1% ones. Certainly, the difference in compositions of the precipitates on the surfaces of the electrodes could undoubtedly distinguish their current responses [9,56].

### 3.5. Corrosion Product Analysis

**Figure 8:**
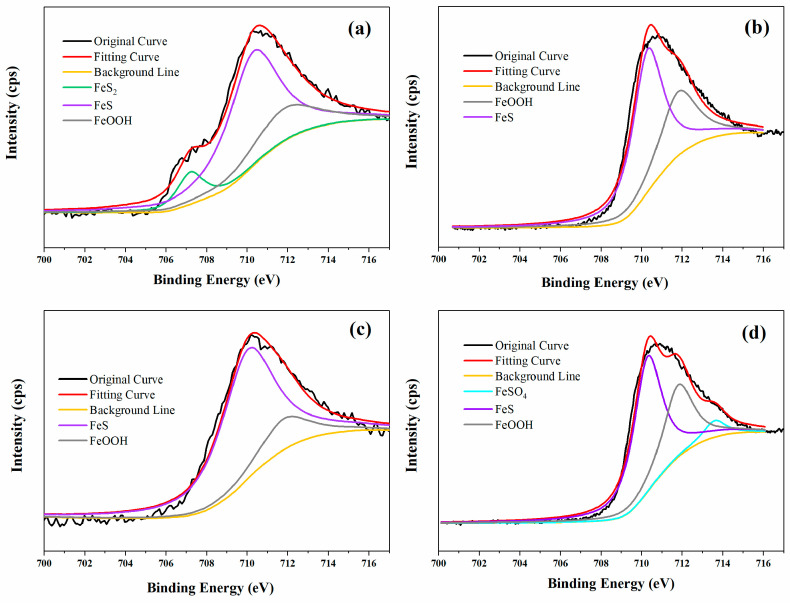
Fe 2p spectra of Q235 carbon steel in (**a**) 100% CS media with SRB, (**b**) 100% CS media without SRB, (**c**) 1% CS media with SRB, and (**d**) 1% CS media without SRB.

Figure 8 shows the high resolution XPS spectra of Fe 2p of coupons in different environments. The results showed that FeS and FeOOH were detected in all the coupons, and the FeS was found to be the main corrosion product in all the specimens, which was a typical corrosion product of the SRB-induced corrosion of steels [13]. The Fe 2p spectra of Q235 steel in 100% CS media with SRB was fitted with three peaks, corresponding to FeS_2_ (707.2 eV), FeS (710.3 eV), and FeOOH (711.8 eV) [57]. For coupons in sterile 1% CS media, the compound of FeSO_4_ (707.2 eV) was also detected in addition to FeS and FeOOH. However, for the coupons in 100% abiotic and 1% CS biotic media, no other compounds were detected except for FeS and FeOOH. Sulfate was reduced to the sulfur ion and then deposited with ferric ions during SRB metabolic activity. The coupons in 1% CS sterile media seemed to have the lowest content of FeS among all the samples, which may be related to the low H_2_S concentration and the absence of bacteria.

**Figure 9:**
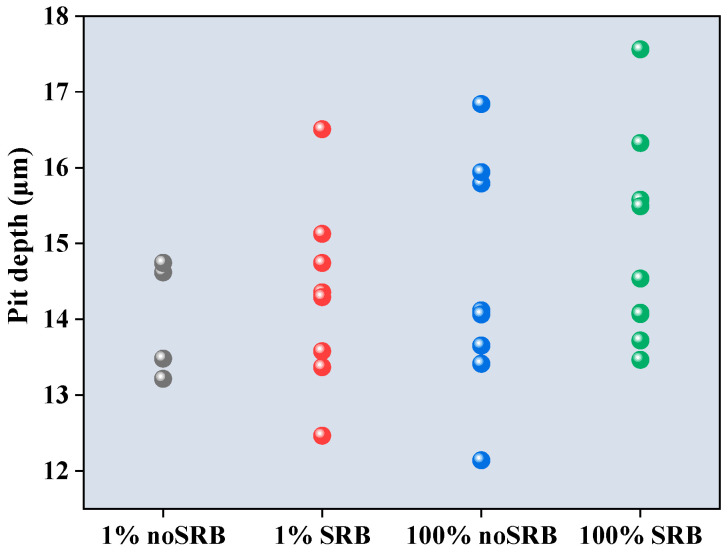
Corrosion pit depth distribution on steel coupons across varied culture media.

The pit depths of steel coupons in different environments were analyzed using a CLSM after removing the corrosion product, as shown in Figure 9. The results showed that the pit depths in 1% CS media without SRB, 1% CS media with SRB, 100% CS media without SRB, and 100% CS media with SRB, were 14.01 ± 0.78, 14.304 ± 1.22, 14.49 ± 1.56, and 14.98 ± 1.36 μm, respectively. The surface roughness (*S*_a_) in 1% CS media without SRB, 1% CS media with SRB, 100% CS media without SRB and 100% CS media with SRB, were 2.76, 2.82, 2.68, and 2.88 μm, respectively. There is no significant difference in the pit depth between 1% CS media with or without SRB (*p* = 0.68), nor is there a significant difference between 100% CS media with or without SRB (*p* = 0.50). In addition, there is no significant difference in the pit depth between 100% CS and 1% CS media with SRB (*p* = 0.30).

## 4. Discussion

MIC proceeds via the interaction between metallic materials, microorganisms, and the environment to facilitate the deterioration of the metal. In this present study, the SRB strain *D. caledoniensis* demonstrated its ability to conduct electrons from polarized electrodes in previous studies [34,35]. The evolution of hydrogen and oxidation might be crucial pathways for the transfer of the electrons between the SRB biofilm and solid electrode. The analyses of the corrosion rate revealed that the effect of EMIC on steel corrosion varied with the varying environments, as well as the growth of microorganisms.

In this present study, the weight loss and electrochemistry results showed that CMIC dominated in both the 100% and 1% CS SRB solutions. The quotient of EMIC to the total MIC is 27.69% and 37.68% in 100% and 1% CS media, respectively. It is not surprising to find that the Q_mean(EMET)_ increases when the carbon sources are limited. However, it was interesting to find that EMIC did not dominate in either 100% or 1% CS system.

This finding is different from the previous report that EMIC dominated in the MIC by Enning [9]. Here, we need to point that both of the culture media and bacteria strain were largely different between this present study and that of Enning [9]. Firstly, it is well known that the culture media play a very important role MIC. The SRB would use lactate but not iron for energy acquisition according to the thermodynamics [9]. In the study of Enning [9], iron acts as the only electron donors, while in this present study, an abundance of CS was supplied.

There is another point that should be noted that there is an inevitable difference in corrosion rate between that valued via weight loss and electrochemical measurements. In addition, the corrosion rate changed with the immersion time, indicating the *Q*_(EMIC)_ varied with time accordingly.

There is no significant difference in the pit depth between 1% CS media with or without SRB (*p* = 0.68), nor is there significant difference between 100% CS media with or without SRB (*p* = 0.50). This indicated that CMIC played a major role in MIC, so the presence of SRB or not caused a certain but insignificant increase in pitting depth. There is also no significant difference in the pit depth between 100% CS and 1% CS media with SRB (*p* = 0.30). This result indicated that the CS starvation does increase the corrosion rate significantly, but the pit depth was not enhanced significantly. This indicated that CMIC might be the primary form of corrosion.

The results obtained in the 1%-CS media indicated that CS starvation would increase the EMIC tendency of SRB, which might correspond to the changes in the metabolic patterns [29]. The bioenergetic theory of MIC indicated that the starved SRB biofilms would switch from organic carbon oxidation into iron oxidation, thereby acquiring energy to maintain metabolism [29]. Sherar et al. [18] observed that SRB generated organic filaments and was potentially interconnected via putative bacterial nanowires in the absence of CS, and the authors suspected that such nanowires were related to the direct electron-conducting process, which also promoted the cathode reaction. The analyses of *Q*_(EMIC)_ in this study revealed that the sessile cells in the biofilms rather than the planktonic ones directly accounted for biocorrosion [46], and the replacement of the medium further confirmed the direct contact between the bacterial cells and electrodes in the electron transport, thus accelerating corrosion.

The contribution of the metabolites to MIC, as well as the stimulation of the cathodic current, was demonstrated by analyzing the results of the abiotic coupons. The effect of the metabolites on MIC might be mainly caused by hydrogen sulfide, which is corrosive, while the stimulation of the cathodic current could be mainly attributed to the soluble mediators in the solution. Microorganisms also secrete several redox compounds during metabolism. These secreted redox mediators are small, diffusible molecules, which act as electron mediators and signaling factors of the metabolic activities of bacteria, as well as biofilm communication [58]. Jian [53] observed that direct electron transfer through the outer membrane, cytochrome c, dominated the mediation of the electron transfer between *Shewanella loihica* PV-4 biofilms and polarized solid electrodes. However, the biocatalytic current decreased after the media were changed, confirming that the soluble redox mediators transferred the electrons between the biofilms and electrode [59]. In this study, it was demonstrated that the secreted electron mediators have contributed to the cathodic reaction and corrosion of the electrode in the abiotic solution by penetrating the membrane.

The LSV measurements were conducted to reveal the relationship between the biofilm and the production of biocatalytic currents in different environments. A similar trend was observed in the cathodic currents of all the coupons in the biotic environments before and after the media change, which confirmed the biocatalytic ability of the attached biofilm in stimulating the cathodic reaction. In addition, the increased bacterial activity in the 10%-CS SRB than in the 100%-CS SRB corresponded to the analyses of MIC. The higher bacterial activity in a modest CS environment leads to more severe corrosion and higher proportion of EMIC in the whole corrosion. Recent studies corroborated the direct contact between bacterial cells and the electrodes to facilitate electron transfer [60], and indicated that cytochrome c and outer membrane enzymes may be involved in the EET process between electrodes and biofilm [19,20,61,62].

EET pathways of the bacteria and metals varied with the environment [63]. All the conclusions are drawn based on the experimental data obtained in the laboratory using the specified culture medium and the H-chamber system. There are certain assumptions in the experiment. For example, it is assumed that the 0.22 μm membrane is not blocked and that all ions except microbe cells can pass through the membrane freely. There also might be some gene expression and regulation in extracellular membrane enzymes and cytochrome c, which would be explored in the next experiment.

## 5. Conclusions

*D. caledoniensis* induced both EMIC and CMIC in Q235 steel. Voltammetric responses revealed that biotic electrodes exhibited significantly higher cathodic currents compared to abiotic electrodes, demonstrating the effective role of attached SRB biofilms in accelerating MIC and enhancing cathodic current. Based on weight loss measurements, EMIC accounted for approximately 27.69% of the total MIC. This proportion varied with the solution environment, increasing to 37.68% when the carbon source concentration in the solution was reduced to 1%. This change was primarily driven by electron transfer facilitated by the biofilm attached to the electrode surface, rather than by metabolites or planktonic cells in the solution. No significant difference in pit depth was observed for Q235 steel in media with or without SRB, nor was pit depth influenced by changes in the carbon source concentration in the solution. The results indicate that *D. caledoniensis* induces MIC in Q235 steel, promoting corrosion, with CMIC being the primary contributor to MIC.

## Figures and Tables

**Table 1:** Electrochemical parameters of the Q235 steels in the sterile, deoxygenated PBS solution after they were immersed in different SRB solutions for 15 days.

Conditions	*R*_s_ (Ohms cm^2^)	*Q_f_*		*R*_f_ (Ohms cm^2^)	*Q*_dl_		*R*_ct_ (Ohms cm^2^)
		*Y*_f_ (×10^−4^ S cm^−2^ S^n^)	*n*_f_		*Y*_dl_ (×10^−4^ S cm^−2^ S^n^)	*n*_dl_	
1% CS with SRB	21.66 ± 1.18	1.12 ± 0.15	0.75 ± 0.35	15.10 ± 2.36	5.79 ± 2.80	0.80 ± 0.034	12,850 ± 212.13
1% CS without SRB	20.46 ± 5.54	3.67 ± 0.78	0.67 ± 0.29	13.72 ± 2.79	2.10 ± 0.97	0.91 ± 0.0078	27,450 ± 70.71
100% CS with SRB	11.34 ± 0.21	64.1 ± 1.17	0.86 ± 0.004	12.20 ± 0.85	14.9 ± 0.80	0.97 ± 0.03	10,410 ± 130
100% CS without SRB	22.22 ± 2.66	23.4 ± 5.04	0.97± 0.03	17.80 ± 0.22	60.7 ± 10.5	0.89 ± 0.05	17,610 ± 390

## Data Availability

The raw data supporting the conclusions of this article will be made available by the authors on request.
